# Seroprevalence of and Associated Risk Factors for Bovine Viral Diarrhea in Dairy Cattle in and Around Nekemte Town, East Wallaga, Oromiya Regional State, Ethiopia

**DOI:** 10.1155/bmri/1709145

**Published:** 2025-01-06

**Authors:** Begna Bulcha, Asaminew Tesfaye, Abebe Garoma, Feyisa Begna

**Affiliations:** ^1^Department of CLiS, School of Veterinary Medicine, Wallaga University, Nekemte, Ethiopia; ^2^Animal Health Institute, Sebeta, Ethiopia; ^3^College of Agriculture and Veterinary Medicine, Jimma University, Jimma, Ethiopia

**Keywords:** bovine viral diarrhea virus, dairy cattle, Nekemte, reproductive problem, risk factors

## Abstract

Bovine viral diarrhea virus (BVDV) is an important pathogen affecting dairy cattle all over the world by causing significant economic losses due to reproductive and respiratory problems, immunosuppressive effects, increased risk of morbidity, and calf mortality. A cross-sectional study was conducted from February 2021 to August 2021 to determine the seroprevalence of bovine viral diarrhea (BVD) and identify risk factors associated with its occurrence in and around Nekemte Town of Ethiopia. Blood samples were collected from 305 dairy cattle of 41 herds by using cluster-sampling method. All sampled animals were identified by their age, breeds, origin, parity, pregnancy status, and history of reproductive and respiratory problems. Competitive ELISA was used in the laboratory to detect the presence of antibodies in the serum. At the animal and herd level, descriptive statistics were utilized to assess the amount of BVDV viral antibody circulation, and multivariable logistic regression analysis was employed to detect potential risk variables. The result demonstrates 9.84% (95% confidence interval (CI): 6.49–13.18) and 28.52% (95% CI: 23.46–33.59) seroprevalence of BVDV antibody at individual and herd level, respectively. Abortion (odds ratio (OR) = 2.75; *p* = 0.019), retention of fetal membrane (OR = 3.33; *p* = 0.011), purchasing of animals (OR = 2.98; *p* = 0.017), and pregnancy (OR = 3.16; *p* = 0.019) were variables significantly associated with the seropositivity of BVDV. Herd size was found to be substantially linked with BVDV infection at the herd level (*p* = 0.009). These moderate seroprevalence of BVDV results indicate that the virus is widely spread among dairy cattle at various farms in and around Nekemte Town, hurting dairy farm production and productivity. To reduce the seroprevalence of this infectious agent, cows with a history of reproductive disorders should be tested, and new animals should be quarantined before being introduced into herds, and more research should be done to assess the impact of reproductive failure and other effects associated with this virus.

## 1. Introduction

Viral infectious diseases are the most known financially important diseases of dairy cattle worldwide [1]. Bovine viral diarrhea (BVD) is a significantly contagious viral disease of cattle inflicting substantial economic losses to the dairy cattle industry worldwide. Its economic losses are the cumulative result of the losses due to morbidity and mortality, an increased premature culling, a decreased reproductive performance, and growth retardation in persistently infected (PI) animals [2–4]. Added to its adverse impact on reproductive, bovine viral diarrhea virus (BVDV) is considered an initiating event for the development of bovine respiratory diseases complex (BRDC), which leads to an increased susceptibility to other diseases due to either its immunosuppressive or synergism with other pathogens [5].

The causal agent of BVD, BVDV, is a member of the *Pestivirus* genus of the Flaviviridae family that is endemic in the bovine populations of numerous nations [6]. It is a tiny (40–60 nm), spherical, positive-sense single-stranded RNA virus with a size of roughly 12.5 kb [7]. Taxonomically, *Pestivirus A* (formerly BVDV-1) and *Pestivirus B* (BVDV-2) are two species, with two biotypes: cytopathic (CP) and noncytopathic (NCP) biotypes, based on the virus's lytic action in cell culture, with NCP BVDV predominating in nature [8, 9].

The disease is found all over the world, and its prevalence varies by continent and country due to many risk factors such as a lack of biosecurity, management systems, environmental differences, herd size, cow population density, and the presence of PI cattle [10]. At individual animal, the major known influencing risk factors reported were herd size, age and breed of animals [11], parity, farm hygiene, introduction of new animal to farm, and production system [12]. The virus is transmitted both vertically and horizontally [13]. The infection may result in persistent infection or transient infection based on the time and route infection. PI animals are key to transmitting BVDV, as they excrete large amounts of the virus throughout their lives and are unable to develop antibodies to the virus. In contrast, transiently infected (TI) animals are less important for disease transmission, as they shed fewer viruses for a brief period (~14 days) [14].

The disease causes substantial costs for farmers through direct and indirect losses [15]. In recent years, the worldwide direct financial losses due to BVDV infection have been estimated to be up to $687.80 per animal per year [16]. The most epidemiologically and economically serious outcome of BVDV infection is the development of PI calves resulting from infection of cattle at the early stage of gestation (2–4 months) [17] before development of the humoral immune system [2]. In spite of the importance of this disease in dairy cattle production, it is not well studied in most sub-Saharan Africa countries including Ethiopia. So far, only certain studies [11, 12, 18–22] have been published in Ethiopia indicating the presence of anti-BVDV antibodies in cattle. In general, seroprevalence has ranged from 8.44% [19] to 80.82% [22], but no information exists on the epidemiological status of the disease in the East Wollega Zone. Assessing the virus's seroprevalence and determining the risk factors associated with illness incidence are critical steps in developing and executing effective control measures. Therefore, the objective of this research was to find more information on the epidemiology of BVDV infections and risk factors associated with BVD occurrence in dairy herds in and around Nekemte Town of Oromia Region, Ethiopia.

## 2. Materials and Methods

### 2.1. Description of the Study Area

**Figure 1:**
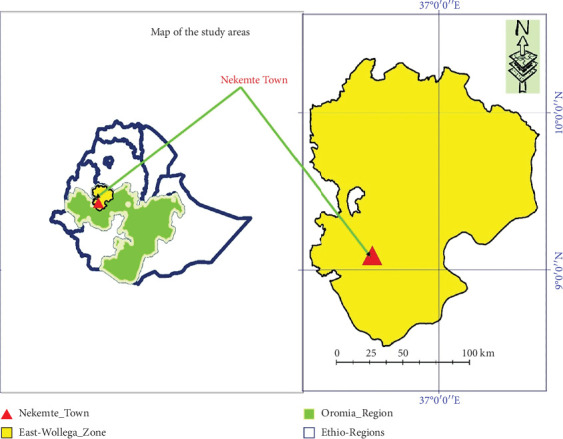
Map of the study areas (created by ArcMap 10.7).

The study was conducted in East Wollega, specifically Nekemte Town and its surroundings. Nekemte Town is located in the East Wollega Zone in the western part of Oromia between 09°03⁣′957⁣^″^–09°06⁣′ 593⁣^″^ N latitude and 36°32⁣′928⁣^″^–36°43⁣′206⁣^″^E longitude and at altitudinal ranges from 2100 to 2250 m above sea level, the highest being Komto area. It is located at about 331 km away from Finfinnee to the western direction possessing a total area of 901.80 km^2^ (Figure 1). The livestock population of the area comprises 74,574 cattle; as received from Guto Gida Woreda Agriculture and Rural Development Office. About 102 commercial dairy farms are there within Nekemte Town of Guto Gida District, and they are used as the main milk shed dairying for the communities of the area. That is, these farms produce milk for household consumption and commercial purposes [23].

### 2.2. Study Population

The study was conducted in dairy cattle reared under intensive and semi-intensive production systems within selected farms. For this study, only unvaccinated dairy cattle above 6 months of age were considered. Local breed and crossbreed (local breed crossed with Holstein-Friesian and Jersey) were breed of animals participating in this study. The study populations were recruited from dairy farms with small (one to 10 animals) and medium (11–25 animals) [11].

### 2.3. Study Design and Sample Size Determination

A cross-sectional study was conducted between February 2021 and August 2021. The number of herds required for the study was determined using the cluster formula described by Ferrari et al. [24] with an assumption of 11.7% [18] expected prevalence of BVD and 10% absolute precision with a 95% confidence level. Herd was considered the primary sampling unit (PSU) whereas the animals selected from each herd were considered the secondary sampling units (SSUs). A total of 41 herds, 21 from small- and 20 from medium farm–sized herds, were selected. Accordingly, about five SSUs and 10 SSUs from small and medium herd were included randomly. Design effect was performed by using a predetermined intracluster correlation coefficient of *ρ* (rho) of 0.42 for BVD [25] to account for the effect of intracluster correlation. The formula used was

 $$\begin{array}{c} N=\frac{Z^{2}*P\left(1-P\right)}{e^{2}}=\frac{1.96^{2}*0.117\;\left(1-0.117\right)}{0.05^{2}} \end{array}$$

Accordingly, 40 study animals were required assuming if simple random sampling effected, but after adjusting for clustering effect, *N*′ = *N* (1 + *ρ*(*m* − 1), 105 animals from small herd and 200 from medium-sized herd were considered, where *N*′ is the new sample size, *N* is the original sample size, and *m* was the number of animals selected from the PSU. Hence, a total of 305 dairy cattle were included.

### 2.4. Sampling and Data Collection

Herds were sampled in cluster sampling technique, and individual dairy cattle were selected with simple random sampling from the selected herds. The putative risk factors include age, breed, parity status, pregnancy status, and history of reproduction problems (abortion, retained fetal membrane (RFM), and stillbirth) at individual animal level. Herd size, farm type, contacts with other herds, breeding methods, management system, and introduction of new animals to herds are data collected at farm level. For this particular study, herd implies a number of animals owned by a single owner.

### 2.5. Blood Sample Collection

Blood samples were collected from individual animals selected randomly as study unit from their jugular vein using plain vacutainer tubes and disposable vacutainer needles. From each animal, about 6–10 mL of blood was collected and kept overnight in an upright position followed by centrifugation at 150 rpm. The serum was decanted into labeled sterile cryovial and stored at −20°C until the laboratory analysis was carried out.

### 2.6. Laboratory Analysis

A blocking ELISA (IDEXX BVDV p80 Ab, Montpellier SAS, France) technique was used to detect an antibody against BVDV according to the manufacturer's instructions. According to the information in the manufacturer's validation data report, the sensitivity (Se) and specificity (Sp) of the ELISA kit used for BVDV were 97.6% and 97.27%, respectively. Any herd with at least one seropositive animal was categorized as a positive herd.

### 2.7. Data Analysis

All the data collected were collated on Microsoft Excel (2016) and transferred to Stata 14.2 (StataCorp, LLC, Texas 77845, United States) for further statistical analysis. The apparent prevalence (AP) of antibody to BVDV was estimated using the proportion of positive results to the total number of cattle examined. True prevalence (TP) was calculated by adjusting the AP for Sp and Se of the test using the formula, TP = AP + (Sp − 1)/Sp + (Se − 1), where Sp is specificity and Se is sensitivity of the test used [26]. Associations between an outcome (anti-BVDV antibodies and explanatory variables) were investigated by using a logistic regression model and Fisher exact test. Adjusted odds ratios (AORs) were used to measure the strength of the association between outcome and explanatory variables. Initially, univariable logistic regression analysis was performed using seroprevalence as the dependent variable. The explanatory variables with a *p* < 0.25 were checked for multicollinearity by using Spearman's correlation coefficient (*ρ*), with *ρ* ≥ 0.6 or ≤ −0.6 as the cut-off points considered for collinearity diagnostic [25]. In a multivariable logistic regression model, variables with a *p* value > 0.05 were removed through a backward elimination procedure, until variables remaining in the final model all reached a *p* value < 0.05 and these variables were be considered potential risk factors.

## 3. Results

In the current study, of the total of 305 tested serum samples of dairy cattle, 30 (9.84%) (95% confidence interval (CI): 6.49–13.18) were positive for antibodies against BVDV. At the herd level, six (30%) (95% CI: 11.89–54.27) medium-sized herds were seropositive animals, but a small farm has no positive animal. The true animal-level seroprevalence estimate was 7.49% (95% CI 3.96–11.01). Considering that at least one positive animal was classified as positive for BVDV in each of the farms, from the 41 cattle herds, the overall herd-level seroprevalence of BVDV was 14.63% (6/41; 95% CI: 5.56–29.17). The seroprevalence of BVDV varies across different variables, like age difference, breed, body condition, parity and pregnancy status, origin of the animal, history of respiratory problems, and diarrhea (Table 1).

According to the current study, the seroprevalence of BVDV was higher in dairy cattle with reproductive disorders (abortion, RFM, repeated breeding, and reduced fertility) than dairy cattle recorded without the mentioned reproductive disorders (Table 2).

In the final multivariable logistic regression model, pregnancy status, abortion, RFM, and origin of the animal were independently associated with BVDV serostatus in the dairy cattle (Table 3). Cows that have an abortion history were 2.75 (odds ratio (OR) = 2.75; *p* = 0.019) times more likely to have BVDV antibodies compared to those that had not had an abortion. Cows with a history of RFM had a higher risk of infection with BVDV than (OR = 3.33; *p* = 0.011) cows without RFM. Concerning pregnancy status, pregnant cows were 3.16 (OR = 3.16; *p* = 0.019) more likely to have anti-BVDV antibodies than nonpregnant dairy cattle. The study also found that animals purchased or introduced into the farm were 2.98 times more likely (OR = 2.98; *p* = 0.017) to have anti-BVDV antibody than homebred cattle.

The result of this investigation indicated that all the positive herds were from a farm with medium herd size (11–25 animals) and none from small herd (5 to 10 animals). The association between herd size and being seropositive to BVDV was significant (*p* < 0.05) with higher prevalence (30%) occurring in a farm with larger herd size (Table 4).

## 4. Discussion

The present study revealed that BVDV is a moderately prevalent pathogen in animals and among dairy farms of Nekemte Town and its vicinity, Oromia Region. The BVDV result obtained at animal level (9.84%) in a current study is comparable with earlier findings of Nigussie et al. [20], Asmare et al. [27], and Asnake et al. [19] who reported 9.59%, 11.7%, and 8.44% prevalence in Jimma Zone, central and southern parts of Ethiopia, and Asella, respectively. The current findings were also in agreement with the previous findings from other countries of the world. For instance, Saeed et al. [28] and Callaby et al. [29] reported a prevalence of 10.7% in Sudan and 10.4% in Egypt and East African countries, respectively.

On the other hand, the current finding was lower compared to animal-level seroprevalence reported elsewhere in Ethiopia including 32.6% [11] in dairy cattle in three milk sheds, 32.9% [27] and 51.9% [12] in Jimma Town dairy cattle, and 26.84% [21] in Gondar City dairy cattle. Likewise, the present finding was lower than other research findings elsewhere in Africa. For instance, seroprevalence of 49.37% in South Africa [30], 30% in Cameroon [31], 40% and 62.2% in Egypt [32], 76.2% in Zambia [33], 66.4% in Nigeria [34], 79.1% and 52.3% in Kenya [35, 36], and 50.4% in Sudan [37] was reported. Difference in anti-BVDV antibody prevalence between different regions and countries might be due to differences in breeds of tested animals, existence of PI animal, type of breeding, management system (introduction of new animal, mixed production with small ruminants), and disease-control measure, and this is important because it indicates the perpetuity in the environment of the virus [38]. In addition, it may be due to the different antigens of BVDV used in serological kits and their cut-off values [39].

However, the seroprevalence estimated in the present study was higher compared to the findings of Talebkhan, Haghparast, and Hajenejad [40] and Manandhar, Yadav, and Singh [39] who reported 3.2% and 2.2% in Nepal and 2.9% [41] and 1.1% [42] in Ivory Coast and Sweden, respectively. The variation in seroprevalence among studies might be due to differences in biosecurity measures, herd size, age of the animals, cattle density, presence of PI animals, and the types of diagnostic test used and sample size considered for the study [31, 43, 44].

In the current study, a 14.63% (6/41) herd-level prevalence of BVDV was recorded. This result is lower compared to herd-level prevalence reported from different parts of Ethiopia by different authors, including 68.3% in Gondar City [21], 22.2% in Asella [19], 95.6% in Jimma Town [12], and 69.8% [11] in other part of the country. This finding is also inconsistent with herd-level prevalence reported in different parts of the world such as 65.5% in Brazil [45], 66% in Great Britain [46], 69% in Colombia [47], 63.1% in Kenya [35], and 92% in Cameron [31]. This could be due the inclusion of small herd size in the current study and might be due to their sampling focused on dairy animals with a history of reproductive disorders in this higher herd prevalence reported. The current moderate to high herd-level seroprevalence recorded may suggest considerable importance of BVDV due to its direct impacts on fertility and neonate mortality and morbidity and indirect effects through immunosuppression and susceptibility to other infections.

In the present study, higher proportions (12.38%) of adult animals (> 18 months) were seropositive compared to younger animals (6–18 months) in which only 2.53% were seropositive. This result concurs with other studies that reported higher prevalence of BVDV antibody in adult age than young age categories [12, 20, 31, 48]. An increase of seroprevalence as age increases is possibly due to an increase in an animal's risk of being exposed to BVDV (cumulative infection with age) [49]. Following transient infection, specific anti-BVDV antibodies can be detected within 3 weeks of infection [50], and animals will remain antibody positive for life, so antibody prevalence reflects the proportion of animals previously exposed to BVDV at any point in life [51]. The lower seroprevalence in young animals could be, at least in part, due to the fact that some of the young animals investigated might be PI animals that are known to be immunotolerant to the virus [52] and do not produce antibodies against the virus to be detected by the ELISA test and can only be detected by antigen ELISA [53, 54].

In the current study, a significantly higher prevalence (*p* = 0.019) of BVDV was found in pregnant cows (OR = 3.16) compared to nonpregnant cows. The finding was consistent with results obtained in previous research [49] in which pregnant cows show higher seropositivity for BVDV. The reason for this might be attributed to peripartum immunosuppression effect [55, 56] that can be associated with the change in the stress hormone (cortisol) level in the body which is well known to increase about a few weeks before parturition [48]. This explanation indicates that animals at the stage of gestation are more susceptible to infection and having the viral infection at this stage may compromise the immunity of the dam and open the way to exposure to opportunistic pathogens [57]. It is better indicated if a longitudinal study with recording of the age gestation is there, which however is not attempted in this study.

In fact, the most important cause of BVDV-associated economic losses appears to be reproductive disorders [11]. The unique ability of pestiviruses to cross the placenta and infect the developing foetus has been shown to lead to a wide array of reproductive losses In line with literature [58, 59] attributing different reproductive problems to BVDV infection, BVDV seroprevalence in the present study was significantly associated with reproductive problems (RFM and abortion).

Accordingly, a highly significant association (*p* = 0.019) was found between cows with abortions and the presence of antibody against BVDV infection. Cows with abortions were 2.75 (OR = 2.75) times more seropositive than those without a history of abortion. The result coincides with other findings [12, 19, 60] who reported a significant association between seropositivity of BVDV and abortion history. These results are also in agreement with Derdour et al. [61] and Thapa et al. [62] which showed that the likelihood of being seropositive to the virus was higher in a cow with abortion history compared to the counterpart. It may be related to the unique ability of pestiviruses to cross the placenta and infect the developing foetus [63]. When a dam becomes TI during early gestation (0–100 days of gestation), infection can result in reduced conception rates and foetal loss, either as a result of early embryonic death, abortion, or absorption [15, 16].

It has been reported that the severe economic impact due to BVDV occurs as a result of repeat breeding, abortion, and neonatal mortality [64, 65]. Scientifically, if exposure and transient infection of the dam occur prior to embryo attachment to the endometrium, infection is avoided, as the BVDV does not penetrate the zona pellucida. However, following attachment, embryonic infection can occur and may lead to embryo loss with the dam returning to heat due to the fetopathogenic effect of BVDV in cattle [66].

The presence of retained placenta was investigated as a complementary factor, which can have an influence on the prevalence of BVDV infection. However, this condition might be complicated by brucellosis, which needs further investigation. Cows with RFM were 3.33 (OR = 3.33) times more likely to have anti-BVDV antibody than cows without the history of RFM. This higher prevalence of BVDV in animals with a history of RFM than in animals without the problem concurs with other studies [60]. The problem of fetal membrane retention or failure to expel fetal membranes within 24 h after parturition is associated with the existence of abortion, dystocia, and impaired neutrophil migration to the placental interference in the periparturient period, which are known sequels of immunosuppression effects of BVD virus infection [67].

In a recent study, the origin of animal whether purchased or homebred was an important risk factor for BVDV (OR = 2.98), which suggests the purchasing of PI animals. This result is in line with many reported from different parts of the world. Talafha et al. [44] have reported that animals that are purchased were more seropositive than those homebred in Jordan. Solis-Calderon, Segura-Correa, and Segura-Correa [68] reported that purchase of cows (introduction of animals to the herd) in small herds increased the prevalence and risk of BVDV infection as compared to middle and large herd sizes. A researcher in Spain dairy cattle found that the seroprevalence of purchased cows was much higher than that of cows whose origin was the farm [38]. However, seropositive animals are not the main risk factor for infection of BVDV, but the presence of PI (seronegative) animals in the herd. This could also be due to the introduction of PI animals, Trojan dams, or contact between animals from infected and noninfected herds which can be transmitting the virus to naive herds [69].

BVDV seroprevalence was found to be high in herds with more than 11 dairy animals. This finding agrees with several studies indicating that BVDV is more prevalent in large herds and in areas with high cattle density [11, 21, 31, 44, 70, 71]. This is justified by the fact that self-clearance is more likely in small herds compared to large herds which commonly have rearing conditions which increase the risk of exposure of PI animals to susceptible seronegative animals in early pregnancy [72].

In this study, almost all of the farmers who introduced new animals to their herds have no isolation room or did not conduct a screening test for BVDV. This was implied as they might have purchased PI animals which are carrier and higher shedders of the virus and facilitate the dissemination of virus within their herds. The use of IDEXX BVDV P80 antibody testing in this study generally revealed the evidence that the BVDV exposure is prevalent in and around Nekemte Town dairy farms, East Wollega. Nonetheless, based on the current study, it is not possible to confirm PI status and tell the genotype of BVDV that might be predominant, whether *Pestivirus 1* (BVDV-1) or *Pestivirus 2* (BVDV-2). Knowing the genotype and subtype of BVDV is very important in terms of control of the infection via vaccination approaches.

## 5. Conclusion and Recommendations

The study confirmed that BVDV is a prevalent and very important disease at individual animal and herd level in and around Nekemte Town dairy farms. The presence of the antibody against this viral infection at herd level indicates that the virus has been circulating as no vaccination has been practiced in the country against it. In relation to the risk factors studied in this particular study, it was indicated that higher seroprevalence of BVDV was estimated in purchased individuals, pregnant cows, and cows with a history of abortion and retained membrane; these factors were found to be potential risk factors associated with seropositivity of BVDV infection at individual animal level. The significant association between reproductive disorders and seropositivity to the viruses revealed their impact on the reproductive and productive performance of dairy cattle and, hence, the profitability of dairy farms in the study area. In general, the results of the current study are essential to understand the epidemiology of the BVDV virus and would be an input to formulate strategies in the country to mitigate the impact of these viruses on the productive and reproductive indicators of cattle farms at the regional or country level. Based on the conclusion, it was recommended that application of stringent biosecurity practices and all purchased cows/heifers should be tested for BVDV and quarantined before mixing with the herd to prevent the introduction or spread of the diseases onto farms.

## Figures and Tables

**Table 1:** Seroprevalence of anti-BVDV antibody analysis across different categorical variables in dairy cattle in and around Nekemte Town dairy farms.

**Variables**	**Categories**	**No. of animals**		**% prevalence (95% CI)**	**Univariable analysis**	
					**Crude OR (95% CI)**	**p** ** value**
		**E**∗	+**v**∗			
Age	Adult	226	28	12.38 (8.39–17.40)	5.11 (4.0–8.34)	0.028
	Young	79	2	2.53 (0.32–8.84)		

Breed	Cross	256	27	10.54 (7.09, 15.03)	0.55 (0.16–1.90)	0.347
	Local	49	3	6.12 (1.28, 16.86)		

BCS	Poor	107	13	12.15 (6.63, 19.87)	1.47 (0.68–3.16)	0.342
	Good	198	17	8.58 (5.08–13.39)		

Parity	Multiparous	136	17	12.50 (7.50, 13.39)	1.93 (0.77–4.86) 1.33 (0.42–4.16)	0.159 0.618
	Primiparous	67	6	8.95 (3.35, 18.47)		
	Noncalving	102	7	6.86 (2.77, 13.50)		

Pregnancy status	Yes	37	8	21.62 (9.82, 38.21)	3.08 (1.25–7.55)	0.014
	No	268	22	8.20 (5.21, 12.16)		

Respiratory	Yes	45	6	13.33 (5.05, 26.79)	(0.58–3.93)	0.396
	No	260	24	9.23 (6.00, 13.42)		

Origin of the animal	Purchased	124	22	17.74 (11.46, 25.62)	4.66 (2.00–10.86)	0.000
	Homebred	181	8	4.42 (1.92, 8.52)		

*Note:E*∗ = number of animals examined; +*v*∗ **=** number of animals that tested positive.

Abbreviation: BCS: body condition score.

**Table 2:** Seroprevalence of BVDV in relation to reproductive problems in dairy cattle in and around Nekemte Town.

**Reproductive problem**	**Status**	**No. of animals examined**	**No. of animals that tested positive**	**% prevalence (95% CI)**	**Univariable analysis**	
					**Crude OR (95% CI)**	**p** ** value**
Abortion	Yes	57	13	22.81 (12.73–35.83)	4.01 (1.82–8.85)	0.001
	No	248	17	6.85 (4.04, 10.74)		
Retained fetal membrane	Yes	40	10	25 (12.69–41.19)	4.08 (1.74–9.53)	0.001
	No	265	20	7.54 (4.67–11.41)		
Repeated breeder	Yes	59	11	18.64 (9.69, 30.91)	2.73 (1.22–6.12)	0.014
	No	246	19	7.72 (4.71, 11.79)		
Infertility	Yes	37	9	24.32 (11.77, 41.19)	3.78 (1.57–9.05)	0.003
	No	268	21	7.84 (4.91, 11.72)		
Stillbirth	Yes	23	3	13.04 (2.77, 28.58)	0.86 (0.19–3.87)	0.849
	No	282	27	9.92 (6.69, 14.03)		

**Table 3:** Multivariable logistic regression analysis of potential risk factors for BVDV antibodies in Nekemte Town dairy farms.

**Variables**	**Categories**	**Multivariable analysis**	
		**Adjusted OR (95% CI)**	**p** ** value**
Retained fetal membrane	Yes	3.33 (1.32–8.42)	0.011
	No		
Pregnancy status	Yes	3.16 (1.20–8.28)	0.019
	No		
Abortion	Yes	2.75 (1.22–6.63)	0.019
	No		
Origin of the animal	Purchased	2.98 (1.21–7.32)	0.017
	Homebred		

**Table 4:** Herd-level seroprevalence of BVDV in and around Nekemte dairy farms.

**Variables**	**Categories**	**Total herd examined**	**No. tested positive**	**% prevalence (95% CI)**	**p** ** value**⁣^∗^
Herd size	Medium	20	6	30 (11.89–54.27)	0.009
	Small	21	0	0.00 (0.0,0.0)	
Breeding methods	AI	34	6	17.64 (6.76–34.53)	0.2835
	Bull	7	0	0.00 (0.0,0.0)	
Production system	Intensive	12	1	8.33 (0.21–38.47)	0.651
	Semi-intensive	29	5	17.24 (5.84–35.77)	
History of abortion	Yes	19	4	21.05 (6.05–45.56)	0.390
	No	22	2	9.09 (1.12–29.16)	
Introduction of new animals	Yes	34	6	17.64 (6.76–34.53)	0.567
	No	7	0	0.00 (−)	

## Data Availability

The data that support the findings of this study are available from the corresponding author upon reasonable request.

## Nomenclature

BVD: bovine viral diarrhea
BVDV: bovine viral diarrhea virus
c-ELISA: competitive enzyme-linked immunosorbent assay
TMB: tetramethylbenzene
PI: persistent infection

## References

[1] Nikbakht G., Tabatabaei S., Lotfollahzadeh S., Nayeri Fasaei B., Bahonar A., Khormali M. (2015). Seroprevalence of bovine viral diarrhoea virus, bovine herpesvirus 1 and bovine leukaemia virus in Iranian cattle and associations among studied agents. *Journal of Applied Animal Research*.

[2] Brodersen B. W. (2014). Bovine viral diarrhea virus infections. *Veterinary Pathology*.

[3] McGowan M., McCosker K., Fordyce G., Kirkland P. (2020). Epidemiology and management of BVDV in rangeland beef breeding herds in northern Australia. *Viruses*.

[4] Wernicki A., Urban-Chmiel R., Stegierska D. (2015). Detection of the bovine viral diarrhoea virus (BVDV) in young beef cattle in eastern and south-eastern regions of Poland. *Polish Journal of Veterinary Sciences*.

[5] Gomez-Romero N., Ridpath J. F., Basurto-Alcantara F. J., Verdugo-Rodriguez A. (2021). Bovine viral diarrhea virus in cattle from Mexico: Current Status. *Frontiers in Veterinary Science*.

[6] Barrett D., Parr M., Fagan J. (2018). Prevalence of bovine viral diarrhoea virus (BVDV), bovine herpes virus 1 (BHV 1), leptospirosis and neosporosis, and associated risk factors in 161 Irish beef herds. *BMC Veterinary Research*.

[7] Booth R. E., Brownlie J. (2016). Comparison of bulk milk antibody and youngstock serology screens for determining herd status for bovine viral diarrhoea virus. *BMC Veterinary Research*.

[8] Ammari M., McCarthy F. M., Nanduri B., Pinchuk L. M. (2010). Analysis of Bovine Viral Diarrhea Viruses-infected monocytes: identification of cytopathic and non-cytopathic biotype differences. *BMC bioinformatics*.

[9] Yeşilbağ K., Alpay G., Becher P. (2017). Variability and global distribution of subgenotypes of bovine viral diarrhea virus. *Viruses*.

[10] Khezri M. (2015). Bovine viral diarrhea (BVD): a review emphasizing on Iran perspective. *Journal of Advanced Veterinary and Animal Research*.

[11] Aragaw K., Sibhat B., Ayelet G., Skjerve E., Gebremedhin E. Z., Asmare K. (2018). Seroprevalence and factors associated with bovine viral diarrhea virus (BVDV) infection in dairy cattle in three milksheds in Ethiopia. *Tropical Animal Health and Production*.

[12] Tadesse T., Deneke Y., Deresa B. (2019). Seroprevalence of bovine viral diarrhea virus and its potential risk factors in dairy cattle of Jimma town, southwestern Ethiopia. *Journal of Dairy, Veterinary and Animal Research*.

[13] Van Roon A. M., Mercat M., van Schaik G. (2020). Quantification of risk factors for bovine viral diarrhea virus in cattle herds: a systematic search and meta-analysis of observational studies. *Journal of Dairy Science*.

[14] Scharnböck B., Roch F. F., Richter V. (2018). A meta-analysis of bovine viral diarrhoea virus (BVDV) prevalences in the global cattle population. *Scientific Reports*.

[15] Pinior B., Garcia S., Minviel J. J., Raboisson D. (2019). Epidemiological factors and mitigation measures influencing production losses in cattle due to bovine viral diarrhoea virus infection: a meta-analysis. *Transboundary and Emerging Diseases*.

[16] Richter V., Lebl K., Baumgartner W., Obritzhauser W., Käsbohrer A., Pinior B. (2017). A systematic worldwide review of the direct monetary losses in cattle due to bovine viral diarrhoea virus infection. *The Veterinary Journal*.

[17] Walz H. L. (2017). *Role of Intrauterine Bovine Viral Diarrhea Virus in Modulating Host Gene Expression at the Maternal-Fetal Interface*.

[18] Asmare K., Regassa F., Robertson L. J., Martin A. D., Skjerve E. (2013). Reproductive disorders in relation to Neospora caninum, Brucella spp. and bovine viral diarrhoea virus serostatus in breeding and dairy farms of central and southern Ethiopia. *Epidemiology & Infection*.

[19] Asnake P., Lemma A., Tesfaye A. (2020). Seroprevalence of Bovine Viral Diarrhea Virus (BVDV) and Its Associated Risk Factors in Dairy Cattle in and Around Assela Town, South East Ethiopia. *PREPRINT (Version 1)*.

[20] Nigussie Z., Mesfin T., Sertse T., Fulasa T. T., Regassa F. (2010). Seroepidemiological study of bovine viral diarrhea (BVD) in three agroecological zones in Ethiopia. *Tropical Animal Health and Production*.

[21] Demil E., Fentie T., Vidal G. (2021). Prevalence of bovine viral diarrhea virus antibodies and risk factors in dairy cattle in Gondar city, Northwest Ethiopia. *Preventive Veterinary Medicine*.

[22] Tesfaye A., Omer A., Hussein A. (2021). Seroprevalence of bovine viral diarrhea virus in local Borana cattle breed and camels (*Camelus dromedarius*) in Ethiopia. *Veterinary Medicine: Research and Reports*.

[23] GWARDO (Guto Gida Woreda Agriculture and Rural Development Office) (2015). *Guto Gida Woreda Agriculture and Rural Development Office, the annual report*.

[24] Ferrari G., Paton D., Duffy S. (2016). *Foot and Mouth Disease Vaccination and Post-Vaccination Monitoring*.

[25] Thrusfield M., Christley R., Brown H. (2018). *Veterinary epidemiology*.

[26] Dohoo I. R., Martin W., Stryhn H. E. (2003). *Veterinary epidemiologic research*.

[27] Asmare K., Sibhat B., Ayelet G., Gebremedhin E. Z., Lidete K. A., Skjerve E. (2018). Serological evidence of bovine herpesvirus-1, bovine viral diarrhea virus and Schmallenberg virus infections in relation to reproductive disorders in dairy cattle in Ethiopia. *Acta Tropica*.

[28] Saeed I. K., Ali Y. H., Taha K. M. (2015). First report of bovine viral diarrhea virus antigen from pneumonic cattle in Sudan. *Journal of Advanced Veterinary and Animal Research*.

[29] Callaby R., Toye P., Jennings A. (2016). Seroprevalence of respiratory viral pathogens of indigenous calves in Western Kenya. *Research in Veterinary Science*.

[30] Njiro S. M., Kidanemariam A. G., Tsotetsi A. M. (2011). A study of some infectious causes of reproductive disorders in cattle owned by resource-poor farmers in Gauteng Province, South Africa. *Journal of the South African Veterinary Association*.

[31] Handel I. G., Willoughby K., Land F. (2011). Seroepidemiology of bovine viral diarrhoea virus (BVDV) in the Adamawa region of Cameroon and use of the spot test to identify herds with PI calves. *PLoS One*.

[32] Selim A. M., Elhaig M. M., Moawed S. A., El-Nahas E. (2018). Modeling the potential risk factors of bovine viral diarrhea prevalence in Egypt using univariable and multivariable logistic regression analyses. *Veterinary World*.

[33] Ghirotti M., Semproni G., De Meneghi D. (1991). Sero-prevalences of selected cattle diseases in the Kafue flats of Zambia. *Veterinary Research Communications*.

[34] Bello S. M., Daneji A. I., Chafe U. M., Abubakar M. B., Jibril A. H., Festus A. (2016). Detection of antibodies to bovine viral diarrhea virus in cattle presented for slaughter at Sokoto metropolitan abattoir, Nigeria. *Journal of Veterinary Medicine and Animal Health*.

[35] Okumu T. A., John N. M., Wabacha J. K., Tsuma V., Vanleeuwen J. (2019). Seroprevalence of antibodies for bovine viral diarrhoea virus, Brucella abortus and Neospora caninum, and their roles in the incidence of abortion/foetal loss in dairy cattle herds in Nakuru District, Kenya. *BMC Veterinary Research*.

[36] Olum M. O., Mungube E. O., Njanja J. (2020). Seroprevalence of canine neosporosis and bovine viral diarrhoea in dairy cattle in selected regions of Kenya. *Transboundary and Emerging Diseases*.

[37] Elhassan A. M., Fadol M. A., El-Hussein A. M. (2011). Seroprevalence of bovine herpes virus-1, bovine herpes virus-4 and bovine viral diarrhea virus in dairy cattle in Sudan. *Pakistan Veterinary Journal*.

[38] Mainar-Jaime R. C., Berzal-Herranz B., Arias P., Rojo-Vázquez F. A. (2001). Epidemiological pattern and risk factors associated with bovine viral-diarrhoea virus (BVDV) infection in a non-vaccinated dairy-cattle population from the Asturias region of Spain. *Preventive Veterinary Medicine*.

[39] Manandhar S., Yadav G. P., Singh D. K. (2018). *Epidemiological survey of bovine viral diarrhoea in dairy cattle in Nepal*.

[40] Talebkhan G. M., Haghparast A. R., Rafati M. S. (2011). The prevalence of bovine viral diarrhea virus in persistently infected cows in industrial dairy herds in suburb of Mashhad-Iran. *International Journal of Veterinary Research*.

[41] Couacy-Hymann E., Bodjo S. C., Koffi M. Y., Danho T. (2007). Observations on rinderpest and rinderpest-like diseases throughout West and Central African countries during rinderpest eradication projects. *Research in Veterinary Science*.

[42] Zimmerli U., Presi P., Heim D. (2009). BVD eradication campaign in Switzerland: first results and outlook. *Schweizer Archiv Fur Tierheilkunde*.

[43] Saa L. R., Perea A., García-Bocanegra I. (2012). Seroprevalence and risk factors associated with bovine viral diarrhea virus (BVDV) infection in non-vaccinated dairy and dual purpose cattle herds in Ecuador. *Tropical Animal Health and Production*.

[44] Talafha A. Q., Hirche S. M., Ababneh M. M., Al-Majali A. M. (2009). Prevalence and risk factors associated with bovine viral diarrhea virus infection in dairy herds in Jordan. *Tropical Animal Health and Production*.

[45] Fernandes L. G., de Campos Nogueira A. H., De Stefano E. (2016). Herd-level prevalence and risk factors for bovine viral diarrhea virus infection in cattle in the State of Paraíba, Northeastern Brazil. *Tropical Animal Health and Production*.

[46] Velasova M., Damaso A., Prakashbabu B. C. (2017). Herd-level prevalence of selected endemic infectious diseases of dairy cows in Great Britain. *Journal of dairy science*.

[47] Ortega D. O., Sarmiento R. A. M., Torreglosa J. C. T., Rocha J. F. (2020). Prevalence and risk factors of bovine viral diarrhea in Colombian cattle. *Veterinary World*.

[48] Sibhat B., Ayelet G., Skjerve E., Gebremedhin E. Z., Asmare K. (2018). Bovine herpesvirus-1 in three major milk sheds of Ethiopia: serostatus and association with reproductive disorders in dairy cattle. *Preventive Veterinary Medicine*.

[49] Daves L., Yimer N., Arshad S. S., Sarsaifi K. (2016). Seroprevalence of bovine viral diarrhea virus infection and associated risk factors in cattle in Selangor, Malaysia. *Veterinary Medicine - Open Journal*.

[50] Nettleton P. (2013). Bovine viral diarrhoea virus: biology, diagnosis and control. *Veterinary Record*.

[51] Houe H., Baker J. C., Maes R. K. (1995). Prevalence of cattle persistently infected with bovine viral diarrhea virus in 20 dairy herds in two counties in central Michigan and comparison of prevalence of antibody-positive cattle among herds with different infection and vaccination status. *Journal of Veterinary Diagnostic Investigation*.

[52] Garoussi M. T., Mehrzad J., Nejati A. (2019). Investigation of persistent infection of bovine viral diarrhea virus (BVDV) in Holstein dairy cows. *Tropical Animal Health and Production*.

[53] Gates M. C., Woolhouse M. E. J., Gunn G. J., Humphry R. W. (2013). Relative associations of cattle movements, local spread, and biosecurity with bovine viral diarrhoea virus (BVDV) seropositivity in beef and dairy herds. *Preventive Veterinary Medicine*.

[54] Shirvani E., Lotfi M., Kamalzadeh M. (2012). Seroepidemiological study of bovine respiratory viruses (BRSV, BoHV-1, PI-3V, BVDV, and BAV-3) in dairy cattle in central region of Iran (Esfahan province). *Tropical Animal Health and Production*.

[55] Patra M. K., Kumar H., Nandi S. (2013). Neutrophil functions and cytokines expression profile in buffaloes with impending postpartum reproductive disorders. *Asian-Australasian Journal of Animal Sciences*.

[56] Walz P. H., Grooms D. L., Passler T. (2010). Control of bovine viral diarrhea virus in ruminants. *Journal of Veterinary Internal Medicine*.

[57] Walz P. H., Chamorro M. F., Falkenberg S. M., Passler T., van Der Meer F., Woolums A. R. (2020). Bovine viral diarrhea virus: An updated American College of Veterinary Internal Medicine consensus statement with focus on virus biology, hosts, immunosuppression, and vaccination. *Journal of Veterinary Internal Medicine*.

[58] Al-Afaleq A. I., Abu-Elzein E. M. E., Al-Khalyfah M. (2007). Severe malformations in calves associated with bovine viral diarrhoea (BVD) virus infection in a dairy cattle herd. *Tropical Animal Health and Production*.

[59] Carlsson U., Fredriksson G., Alenius S., Kindahl H. (1989). Bovine virus diarrhoea virus, a cause of early pregnancy failure in the cow. *Journal of Veterinary Medicine Series A*.

[60] Uddin M. A., Ahasan A. S. M. L., Islam K. (2017). Seroprevalence of bovine viral diarrhea virus in crossbred dairy cattle in Bangladesh. *Veterinary World*.

[61] Derdour S. Y., Hafsi F., Azzag N. (2017). Prevalence of the main infectious causes of abortion in dairy cattle in Algeria. *Journal of Veterinary Research*.

[62] Thapa A., Acharya M. P., Raut R., Rimal S. (2019). Seroprevalence and Risk Factors of Bovine Viral Diarrhea in Improved Cattle of Chitwan, Nawalpur and Rupandehi Districts of Nepal. *Nepalese Veterinary Journal*.

[63] Evans C. A., Pinior B., Larska M. (2019). Global knowledge gaps in the prevention and control of bovine viral diarrhoea (BVD) virus. *Transboundary and Emerging Diseases*.

[64] Mahmoud M. A., Allam A. M. (2013). Seroprevalence of bovine viral diarrhea virus (BVDV), bovine herpes virus type 1 (BHV-1), parainfluenza type 3 virus (PI-3V) and bovine respiratory syncytial virus (BRSV) among non vaccinated cattle. *Global Veterinaria*.

[65] Thobokwe G.

[66] Sibel G. Ü. R. (2011). Prevalence of bovine viral diarrhoea, bovine herpesvirus type 1 and 4 infections in repeat breeding cows in Western Turkey. *Brazilian Journal of Veterinary Research and Animal Science*.

[67] Ridpath J. (2010). The contribution of infections with bovine viral diarrhea viruses to bovine respiratory disease. *Veterinary Clinics: Food Animal Practice*.

[68] Solis-Calderon J. J., Segura-Correa V. M., Segura-Correa J. C. (2005). Bovine viral diarrhoea virus in beef cattle herds of Yucatan, Mexico: seroprevalence and risk factors. *Preventive Veterinary Medicine*.

[69] Segura-Correa J. C., Zapata-Campos C. C., Jasso-Obregón J. O., Martinez-Burnes J., López-Zavala R. (2016). Seroprevalence and risk factors associated with bovine herpesvirus 1 and bovine viral diarrhea virus in North-Eastern Mexico. *Open Veterinary Journal*.

[70] Almeida L. L., Miranda I. C. S., Hein H. E. (2013). Herd-level risk factors for bovine viral diarrhea virus infection in dairy herds from Southern Brazil. *Research in Veterinary Science*.

[71] Humphry M. J., Kraus B., Hurst A. C., Maiden A. M., Rodenburg J. M. (2012). Ptychographic electron microscopy using high-angle dark-field scattering for sub-nanometre resolution imaging. *Nature Communications*.

[72] Radostits O. M., Gay C. C., Hinchcliff K. W., Constable P. D. (2007). A textbook of the diseases of cattle, horses, sheep, pigs and goats. *Veterinary Medicine*.

